# Influence of different veneering techniques and thermal tempering on flexural strength of ceramic veneered yttria partially stabilized tetragonal zirconia polycrystalline restoration

**DOI:** 10.4317/jced.55722

**Published:** 2019-05-01

**Authors:** Niwut Juntavee, Pitsinee Serirojanakul

**Affiliations:** 1 Department of Prosthodontics, Faculty of Dentistry, Khon Kaen University, Khon Kaen, Thailand; 2 Division of Biomaterials and Prosthodontics Research, Faculty of Dentistry, Khon Kaen University, Khon Kaen, Thailand

## Abstract

**Background:**

Different technique for ceramic veneering and thermal tempering process are expected to be a reason for alteration in strength of ceramic veneered zirconia. This study evaluates the effect of different veneering technique and varied thermal tempering process on flexural strength of ceramic veneered zirconia.

**Material and Methods:**

Ceramic veneered zirconia bars (25 mm length, 4 mm width, 0.7&1.0mm of zirconia & ceramic thickness) were prepared from zirconia block (e.max® ZirCAD), sintered at 1500°C for 4 hours, and veneered with ceramics with different techniques including CAD-fused using e.max CAD® (C), Pressed-on using e.max® Zirpress (P), and layering using e.max® ceram (L), with different tempering process through fast (F), medium (M), and slow (L) cooling (n=15). The specimens were determined for flexural strength on a universal testing machine. ANOVA and Bonferroni’s multiple comparisons were used to determine for significant difference (α=0.05). Weibull analysis was applied for survival probability, Weibull modulus (m), and characteristics strength (σc). The interfaces were microscopically examined. The phase transformation of zirconia was determined using X ray diffraction.

**Results:**

The mean±sd (MPa), m, σc of flexural strength were 922.06±83.45, 12.78, 958.32 for CF, 924.26±74.64, 14.28, 959.62 for CM, 930.25±92.42, 11.83, 970.83 for CS, 518.29±59.97, 10.11, 542.97 for PF, 516.50±67.51, 8.75, 539.17 for PM, and 520.51±42.38, 14.59, 544.51 for PS, 604.36±64.09, 11.28, 630.67 for LF, 583.81±56.95, 11.67, 609.81 for LM, 547.33±52.23, 12.19, 569.36 for LS. The flexural strength was significantly affected by veneering technique (*p*<0.05). No significant effect on flexural strength upon tempering process was evidenced (*P* >0.05). Phase transformation from t→m related with veneering and tempering procedure.

**Conclusions:**

Strength of ceramic veneered zirconia associated with different veneering techniques, but not directly related with tempering process. CAD-on ceramic veneering zirconia is benefit for enhancing the strength of ceramic bilayer and was recommended as a method for ceramic veneering zirconia.

** Key words:**CAD-CAM, cooling process, flexural strength, thermal tempering, zirconia.

## Introduction

The increasing demand for highly esthetic and predictably long-lasting restorations has led to the development of several ceramics for restorative dentistry, based on computer-aided design and computer-aided manufacturing (CAD-CAM) technology (1). The CAD-CAM fabricated ceramic restorations provide reliable strength, since they are constructed from prefabricated ceramic blanks that possess minute amounts of pores and impurities, leading to ceramic failure. Particular dental ceramics have been increasingly developed for CAD-CAM restoration, encompassing yttria partially stabilized zirconia polycrystalline (Y-TZP), due to its metastatic phase transformation that is capable of enhancing strength and fracture toughness (2). The suitability of Y-TZP for extensive all-ceramic restoration related with marginal fidelity has been described (3). The relatively opaque white color of Y-TZP requires translucence veneering ceramic to produce a natural tooth appearance. Veneering ceramics usually possess low fracture toughness, thus becoming the weak part of the restorations. Chipping and delamination of veneering ceramic from Y-TZP was frequently described as creating frustration for clinicians and patients (4). The failure rate of ceramic was reported as 36% for zirconia-based restoration, commonly related with chipping, compared to 16% for metal ceramic restoration (5,6). Failure of ceramic veneered Y-TZP was related to substructure design, veneering ceramic, coefficient of thermal expansion (CTE) mismatch, residual stress, number of firing, and veneering methods (7-9).

Ceramic-veneered zirconia is generally constructed through the traditional layering technique, using feldspathic porcelain or other glass ceramics, for instance nano-fluoroapatite-, leucite reinforced-, and lithium disilicate-glass ceramic (10). These ceramics need to possess a CTE compatible with zirconia in order to achieve a durable bond and enhance reliable strength of the restoration (11). The porcelain powder is mixed with modeling liquid, applied on zirconia, and fired for a minimum of three firing cycles to derive for the final anatomy, which mostly depends on the skill of the dental technicians. This procedure is time-consuming. Ceramic-veneered zirconia can be constructed through the press-on technique, in which the restorations need to be waxed into the final anatomical contours on the zirconia, and invested in the investment. The investment mold is then burnt out to eliminate the wax, and further heat-pressed with a ceramic ingot. The press-on technique has become increasingly popular due to the fabrication process, which offers some advantages in term of speed, accuracy, and precise anatomy (12). However, the behavior of the pressed ceramic-veneering zirconia towards the cooling method is likewise ambiguous because the cooling phase after pressing is achieved slowly inside the investment mold, and may be different from the cooling process during glazing. Nowadays, CAD-CAM generated veneering ceramic can be merged to the CAD-CAM generated zirconia by a fusion technique (CAD-on), using a fusing glass (13). This technique seems to be reliable compared to others, since the fabrication process is based on digital technology, using industrialized blanks for both veneering ceramic and zirconia.

The strength of ceramic-veneered zirconia is affected by the thermally related sintering procedure. The sintering process is a thermally generated crystallization of veneering ceramic to be adhered to the zirconia substructure. The promptly applied heat needs to be radiated from the muffle of the sintering furnace to reach the external surface of restoration and properly conducted through the inner surface to derive for suitable maturation of ceramic. Similarly, the veneering ceramic and zirconia substructure need to release the heat from the matured stage to room temperature (RT) during the cooling phase as a tempering procedure. This stage generates a thermal effect and crucially developed residual stress, affecting the strength of the restoration. The tempering process directly relates to the rate and method of cooling from the sintering temperature to RT, which induces appropriate residual stress in the ceramic-veneered zirconia that directly affects the reliability of strength (14). Previous studies have shown that the amount and type of residual stress was influenced by veneering technique (15,16). Accumulated residual stresses during the cooling process of ceramic veneering have recently been presented as a major cause of veneer chipping (17-19). Some studies advise slow cooling because fast cooling introduces greater residual tensile stress in the veneering ceramic and generates tensile stress to initiate crack propagation (20,21). The slow cooling may reduce the thermal gradient, producing appropriate residual stress, since it increases time for plastic flow state (22). Other studies reported better flexural and bond strength for ceramic-veneered zirconia upon fast cooling (22,23).

The residual stresses are generated as a result of the thermal gradient from the CTE difference between zirconia and veneering ceramic during the cooling period until the glass transition temperature (Tg) is reached (14). Such condition increases the possibility of ceramic chipping and fracture (24). It was recommended that veneering ceramic should possess CTE for approximately 0.77–0.87×10−6/ oC lower than zirconia to provide suitable residual compressive stress to facilitate a favorable bond and confer strong ceramic-veneered zirconia (11,25). Excessive residual stress also developed from the difference in thermal gradient during ceramic transformation from viscoelastic to solid stage, causing failure of ceramic-veneered zirconia (26). The fact that zirconia possesses low thermal diffusion property means its capability of heat conduction upon sintering is less than that of veneering ceramic. Therefore, the heat accumulation in the ceramic-veneered zirconia plays a significant role in strength (27,28). Although clinicians have chosen to use veneering ceramic with compatible CTE to zirconia, extensive residual stresses still develop. Controversies still exist concerning the behavior of the veneering method and tempering process on the strength of the restoration. Appropriated veneering and tempering methods would provide significant benefits for a clinician in selecting a durable restoration for fabrication. This study is aimed at investigating the influence of different tempering procedures and veneering techniques on flexural strength ceramic-veneered zirconia. It was hypothesized that varying the tempering process to different veneering techniques did not significantly influence flexural strength.

## Material and Methods

The specimens for evaluation of flexural strength of ceramic veneering Y-TZP ( Table 1) were prepared according to three veneering techniques, Cad-on (C), Press-on (P), and conventional layering (L), in relation with three different thermal tempering processes, achieved through fast (F), medium (M), and slow (S) cooling procedures.

**Table 1:**
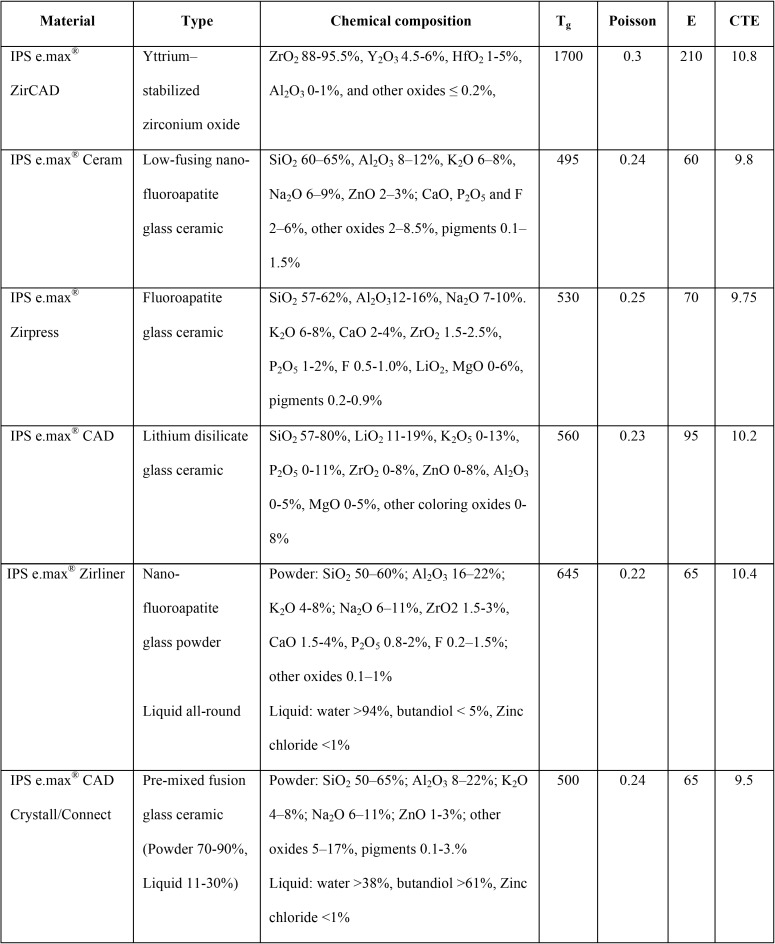
Chemical composition (% by weight), glass transitional temperature (Tg, oC), Poisson’s ratio (ν), elastic modulus (E, GPa), coefficient of thermal expansion [CTE; (X10-6/ oC)] of ceramic materials used in this study.

-Preparation of Zirconia Specimen

A hundred and thirty-five Y-TZP (IPS e.max® ZirCAD, Ivoclar Vivadent, Schaan, Liechtenstein) bars [width (W), length (L), thickness (T) = 5, 31.25, 0.9 mm) were prepared using sectioning apparatus (Isomet® 1000, Buehler, IL, USA), and fired in the furnace (inFire® HTC speed, Sirona, Bensheim, Germany) at the recommended temperature of 1500ºC for four hours, using a heat rate of 5 ºC/min, to derive for fully sintered bars (W, L, T = 4.0, 25, 0.7 mm) due to 20% sintering shrinkage. All bars were randomly segregated into nine groups (15 bars each) to be veneered with ceramics (W, L, T = 4, 25, 1.0 mm) according to three different veneering and tempering methods.

-Conventional Ceramic Layering Technique

A thin layer of special liner (IPS e.max Zirliner, Ivoclar-Vivadent) was applied to the zirconia bars, which were sintered in a furnace (Programmat® P100, Ivoclar Vivadent) twice to reach a thickness of 0.1 mm. Then, the creamy mixed consistency of dentine ceramic (IPS e.max ceram, Ivoclar-Vivadent) was applied, condensed with ultrasonic condenser (3M Unitek, St. Paul, MN, USA), and fired for no more than three times to derive for the final dimensions.

-Press-on Ceramic Veneering Technique

The zirconia bars were applied with a thin layer of special liner and fired in a furnace as previously described. The wax pattern was applied on the sintered liner surface of zirconia using blue inlay wax (Kerr, Orange, CA, USA), invested in the investment mold (PressVest speed, Ivoclar-Vivadent), and then heat-pressed with pressable ceramic (IPS e.maxZirpress, Ivoclar-Vivadent) at 910ºC in the pressing furnace (Programmat EP500, Ivoclar-Vivadent). After divestment, the samples were finished and polished to derive for the final dimension.

-CAD-on Ceramic Veneering Technique

The bar-shaped veneering ceramics (W, L, T = 4.0, 25.0, 1.0 mm) were prepared from pre-crystallized lithium disilicate blocks (IPS e.max CAD HT, Ivoclar-Vivadent) using a precision machine. The zirconia bar and ceramic bar were joined with the Crystall/Connect (Ivoclar Vivadent), and fired at 840ºC as per manufacturer’s instruction.

-Thermal Tempering Process

The specimens were subjected to different tempering processes through F-, M-, and S-cooling. F-cooling was performed by fully opening the furnace’s muffle after sintering to allow for cooling to RT. M-cooling was performed by leaving the specimen to cool down in the closed muffle until Tg of each veneering ceramic was reached, and then fully opening the muffle to cool to RT. S-cooling was performed by leaving the specimen to cool in the closed muffle until a temperature of 200 ºC, and then fully opening the muffle to cool down to RT.

-Evaluation for Flexural Strength

**Figure 1:**
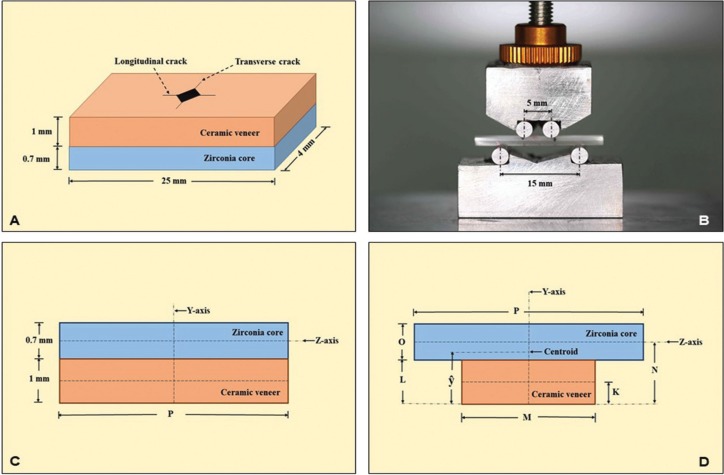
Indentation crack was induced on the surface of veneering ceramic of the ceramic bilayer (A). The bilayer ceramic was compressively loaded on four point bending apparatus (B). The composite beam of ceramic veneering zirconia (C) was transformed to a uniform beam of zirconia (D).

Controlled cracks were initiated on the veneering ceramic surface with Vickers diamond indentation of the microhardness tester (FM800, Future-tech, Tokyo, Japan) at 4.9 N load, for 15 seconds dwelling time, to produce transverse and longitudinal crack lines, and optically measured for crack length (×50 magnification) at 24 hours after initiation (Fig. 1A) to permit for absolute crack propagation caused by environment mortification and surrounding stress. All samples were kept in dry stage at RT prior to evaluation for flexural strength. The specimen was placed, with the veneering ceramic on the tension side, on the four-point bending apparatus, with 15 mm for the outer span (Lo) and 5 mm for the inner span (Li), with a universal testing machine (Lloyd, Leicester, UK). The sample was compressively loaded until fracture at 0.5 mm/min crosshead speed (Fig. 1B). The fracture load (F) was recorded and computed for fracture strength (σf) based on the composite beam theory through a transformation principle. The transformation factor (n) was calculated from the ratio of the elastic modulus of the zirconia core (Ec) to the elastic modulus of veneering ceramic (Ev) as shown in equation 1. The actual beam width (P) for veneering ceramic was transformed to a uniform width (M) of Y-TZP by using equation 2. The transformation of a composite beam comprising Y-TZP and veneering ceramic (Fig. 1C) was transformed to a uniform beam of Y-TZP (Fig. 1D). Then, the centroid (ŷ) of the transformed beam was determined from equation 3. The maximum moment (τm) and moment of inertia (I) were calculated from equations 4 and 5 respectively. The flexural strength (σf) for the transformed beam was determined by equation 6. Then, the given stresses in the transformed beam were converted into flexural strength in the actual composite beam by dividing by the transformation factor.

In which: ŷ: perpendicular distance from centroid to bottom of transformed beam, τm: maximum moment,

I: moment of inertia, Ff: failure load, σf: flexural strength, Lo: outer span = 15 mm, Li: inner span = 5 mm

-Microscopic Examination

The specimens in each group were microscopically examined on the fracture surface and at the ceramic-veneered zirconia interface. The specimens were rinsed with water, dehydrated with acetone, and sputter-coated with palladium-gold using coating apparatus (Emitech K-500X, Asford, British, England) with vacuum 130 m-torr, at 10 mA current, for three minutes. The fracture surfaces and zirconia-ceramic interface of specimens for each group were determined for scanning electron microscopy (SEM, S-3000N, Hitachi, Tokyo, Japan) and energy dispersive analysis of x-ray (EDAX, Oxford Co., Oxfordshire, UK) for characterizing the failure surface and interfacial zone.

-Evaluation of Crystal Structure

The crystal phases of ceramic-veneered Y-TZP were determined for the quantity of tetragonal (t) and monoclinic (m) crystalline structures of Y-TZP with the X-ray diffractometer (XRD, PW 1830, Philips, Almalo, Netherland). The specimens were scanned with copper k-alpha (Cu Kα) radiation from 2-theta (θ, degree) of 20–40o with 0.02o stepwise for two-second intervals. The crystalline phase was determined and compared to the standard database on powder diffraction, and calculated for respective d-values with Bragg equation 7 (29).

In which: λ: wavelength (0.154058 nm for CuKa), d: distance of planes in the hkl-Miller indices, θ: Bragg angle

The proportion of m- to t- crystal was quantified from the spikes’ magnitudes using X-Pert Plus software (Philips Co., Almelo, Netherland). The amount of m-phase to overall zirconia phase content was computed from Garvie-Nicholson equation and further adjusted for nonlinearity with the Toraya equation, as shown in equations 8, 9, and 10 (30).

In which: It and Im: essential intensities of t- and m- phase, C: compositional corrected factor (C = 1.32), Xt and Xm: the Toraya-corrected portion of t- and m- phase of Y-TZP

-Statistical Determination

The data was statistically evaluated with software SPSS/PC+ Version-20 (IBM Corp., Armonk, NY, USA). An analysis of variance (ANOVA) was determined for significant differences of fracture strength relative to the ceramic veneering method and tempering procedure. Bonferroni post-hoc multiple tests were evaluated for the difference between variables at α=0.05. An analysis of fracture strength’s reliability was determined by Weibull++®statistics (Relia-Soft, Tucson Co., AZ, USA), and appraised for Weibull modulus (m) using equation 11 in conjunction with the slope of graph sketched between ln{ln(1/Ps(Vo))} and m ln(σ/σo).

Where: Ps (Vo): survival probability of identical sample, Vo: volume of sample, σf: flexural strength; σo: characteristic flexural strength, m: Weibull modulus.

## Results

**Figure 2:**
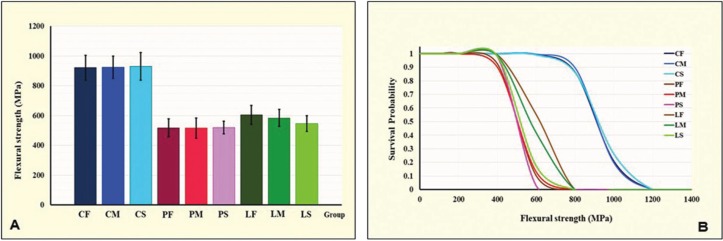
(A) Comparison of flexural strength, and (B) Weibull survival probability for each group of zirconia veneered with veneering ceramic through CAD-fused (C-), Pressed-on (P-), and Layering (L-) technique, and tempered upon fast (F-), medium (M-), and slow (S-) cooling process.

The mean, standard deviation (s.d), 95% confidence interval (CI), Weibull modulus (m), characteristic flexural strength, and relative phase transformation for every group is demonstrated in  Table 1, Table 2 and Figure 2(A). The maximum flexure strength (mean±sd, MPa) was indicated for CS-group (930.25±92.42), followed by CM (924.26±74.64), CF (922.06±83.45), LF (604.36±64.09), LM (583.81±56.95), LS (547.33±53.23), PS (520.51±42.38), PF (518.29±59.97), and PM (516.50±67.51). The Weibull’s modulus (m) for CF, CM, CS, PF, PM, PS, LF, LM, and LS were 12.78, 14.28, 11.83, 10.11, 8.74, 14.59, 11.28, 11.67, and 12.19, respectively. The ANOVA revealed a statistically significant difference in flexure strength due to the different veneering techniques (*p*<0.05). No statistically significant differences were indicated on mean flexural strength due to the effect of the tempering process and the interaction between these two factors (*p*>0.05) as presented in  Table 3. Bonferroni multiple tests suggested a significant difference in flexural strength among different veneering techniques (*p*<0.05). The C-veneering technique provided significantly higher flexural strength than L- and P-veneering (*p*<0.05). However, the tempering process did not initiate a significant impact on flexural strength ( Table 4). The CAD-on provided a higher impact on flexural strength compared to other veneering techniques (*p*<0.05). No significant differences in flexure strength were indicted among ceramics fabricated from either P- or L- veneering technique upon different tempering processes (*p*>0.05), except for the LF group, which was significantly different from PF, PM, and PS (*p*<0.05) ( Table 4). The characteristic flexural strengths (σo, MPa) ranking from highest to lowest were CS (970.83), CM (959.32), CF (958.32), LF (630.67), LM (609.81), LS (569.36), PS (544.51), PF (542.97), and PM (539.17), which indicated the probability of survival upon flexure strength among groups as manifested in Figure 2(B).

**Table 2:**
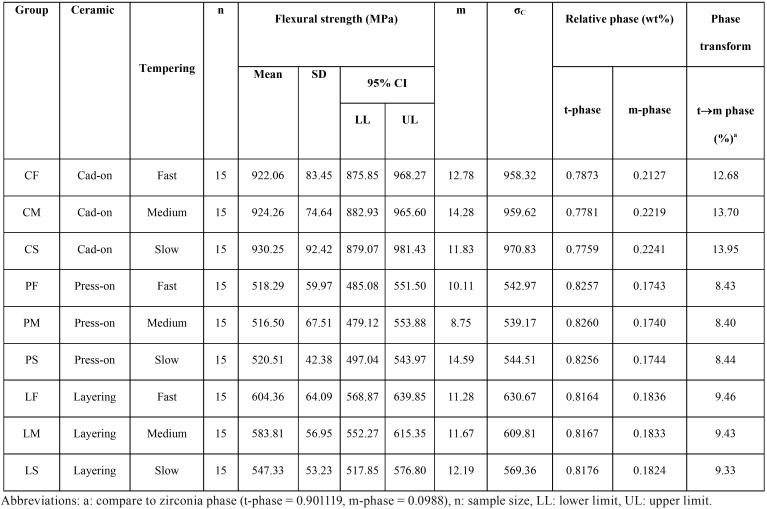
Mean, standard deviation (SD), 95% confidential interval (CI), Weibull’s modulus (m), characteristic strength (σC), relative phase content (wt%), and percentage of phase transformation (%) for flexural strength (MPa) of ceramic veneered zirconia with cad-on (C-), press-on (P-), and layering (L-) technique upon tempering process based on fast- (F-), medium- (M-), and slow- (S-) cooling procedure.

**Table 3:**
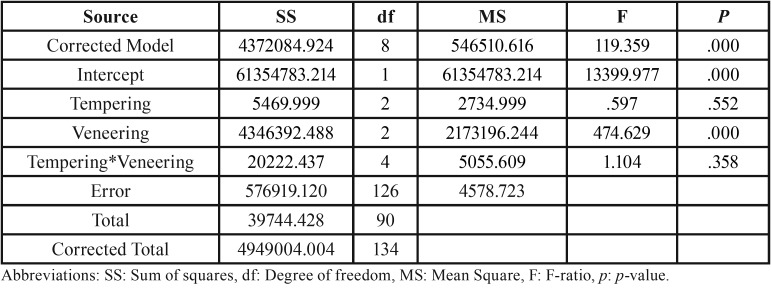
An analysis of variance (ANOVA) of flexural strength for ceramic veneered zirconia upon different veneering and tempering process.

**Table 4:**
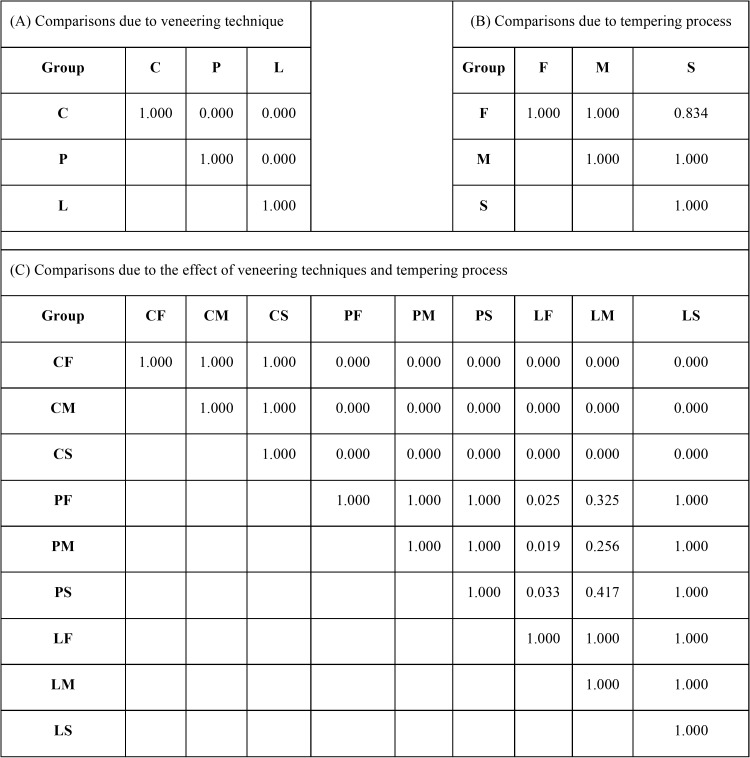
Bonferroni multiple comparisons of flexural strength of ceramic veneered zirconia due to the effect of veneering techniques including cad-on (C-), press-on (P-), and layering (L-) technique (A), tempering process based on fast (F-), medium (M-), and slow (S-) cooling procedure (B), and interaction of the veneering technique and tempering process (C).

**Figure 3:**
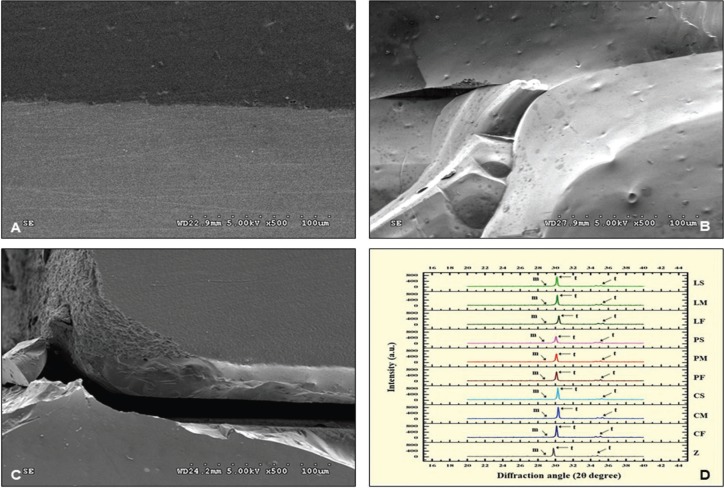
(A) SEM photomicrograph (X 500) demonstrated harmonized interface of ceramic veneering zirconia. (B) The fracture pattern initiated from the indentation crack, and propagated through the ceramic veneering zirconia (C). (D) The XRD patterns indicated different amount of tetragonal (t-) and monoclinic (m-) phase in each group of zirconia veneered with veneering ceramic through CAD-fused (C-), Pressed-on (P-), and Layering (L-) technique, and tempered upon fast (F-), medium (M-), and slow (S-) cooling process.

The SEM-micrographs revealed a harmonized inter-digitation between zirconia and veneering ceramic for every group, which indicated favorable bonding of ceramic-veneered zirconia upon different veneering techniques and tempering processes (Fig. 3A). However, the veneering ceramic revealed several porosities in the layering technique and quite fewer in the pressing technique, while almost absent in CAD-on groups. The photomicrograph revealed the fracture patterns that initiated from the indentation crack and propagated through the veneering ceramic and zirconia substructure as shown in Figure 3(B). The fracture path originated from the tension surface of veneering ceramic, rapidly propagated through the zirconia, and resulted in catastrophic failure (Fig. 3C). The fracture propagation for the CAD-on group was almost perpendicular with the zirconia core, whereas the others were obliquely propagated.

The XRD patterns mostly indicated t-crystalline phase and a minimal amount of m-crystalline phase for each group as indicated in Figure 3(D). The dominant peaks of t-crystalline phase were observed upon the diffraction angle (2θ, degree) of 30.29° that correlated with the 101-crystalline plane. The other peaks of t-crystal phase were discovered at the diffraction angle of 34.57° and 35.271° that related with the 002- and 110-crystallographic plane of the t-phase, as indicated on the XRD-micrograph of zirconium oxide. The minor m-crystal phases were discovered at the diffraction angle of 28.75° and 34.75°, which corresponded to the monoclinic (111) and monoclini (11ī) crystallographic plane. The relative concentration (wt.%) of m-phase regarding the total zirconia phase varied in the amount of phase transformation from t→m phase due to the difference in veneering techniques and tempering processes ( Table 2). The t→m phase transformations were 12.68% for CF, 13.70% for CM, 13.95% for CS, 8.40% for PF, 8.43% for PM, and 8.44% for PS, 9.46% for LF, 9.43% for LM, and 9.33% for LS. The amount of t→m phase transformation was relatively associated with flexure strength upon different veneering techniques and tempering processes.

## Discussion

In this investigation, the flexure strength of ceramic-veneered zirconia was influenced by the veneering technique but not disturbed by the tempering process. Thus, a null hypothesis was rejected for the effect of the ceramic veneering technique, and accepted for the effect of the tempering process. The CAD-on veneering technique significantly engendered higher flexural strength for ceramic-veneered Y-TZP than other veneering techniques as supported by other studies (2,27). The CAD-on technique is capable of establishing higher flexural strength than others may relate with three factors. Firstly, the CAD-on technique utilized lithium disilicate glass ceramic as a veneering material, while the press-on technique used fluoroapatite glass ceramic and the layering technique used low fusing nano-fluoroapatite. The lithium disilicate ceramic possesses stronger fracture resistance (350–400 MPa) than the other fluoroapatite glass ceramics (90-110 MPa). Thus, the different in strength of veneering ceramic relatively influences the strength of ceramic-veneered zirconia (2). Secondly, the CAD-on technique utilized ceramic blanks to fabricate veneering and further sintered to the zirconia core, resulting in a high density of veneering ceramic, enabling flexural strength enhancement. Conversely, the press-on technique used a ceramic ingot to be melted and casted onto zirconia. The pressing procedure created a number of porosities and inclusions inside the pressed ceramic and at the interface, leading to compromised flexural strength. The porosities initiated a negative impact on elastic properties and strength. They acted as stress concentrators to amplify tensile stress failure. Although an annealing process could eliminate some porosity, the residual pores still remain. These pores were found in all veneering ceramic fabricated from layering and the press-on technique, but rarely observed in the CAD-on technique as evidenced in the SEM. Moreover, the effect of sandblasting used during the ceramic divesting process of the press-on technique could induce surface flaws, which was the origin of the crack and compromised strength. Lastly, the impurities incorporated at the ceramic-zirconia interface had a significant impact on ceramic-zirconia bonding quality and eventually affected the strength. The press-on technique was microscopically evidenced with more impurities incorporated at the interface than others; thus, the lowest flexural strength was indicated for the pressed-on, as supported by other studies (26,27).

The thermal tempering processes indicated no significant effect on flexural strength. However, it seems to demonstrate that prolong tempering through S-cooling exhibited a higher impact on enhancing flexural strength than M- and F-cooling for both CAD-on and press-on techniques. Conversely, a short tempering process through F-cooling exhibited higher flexural strength than M- and S-cooling for the L-veneering technique. This probably indicated that S-cooling for the CAD-on technique was capable of inducing appropriated residual stress to enhance flexural strength as supported by another study (2). This was in agreement with higher t→m phase transformation for S-cooling than M- and F-cooling for both CAD-on and press-on techniques as well as higher t→m phase transformation for F-cooling than M- and S-cooling for layering technique. The amount of t→m phase transformation was capable of enhancing the ceramic-zirconia bond strength which eventually strengthened ceramic as supported by other studies (2,6,8,11). In this study, the Tg was used as a reference in setting the cooling process, in which the muffle was open above, at, or below the Tg of veneering ceramic. All specimens were fired according to the cooling regimens for both sintering and glazing processes, and left in the muffle until the ambient temperature was reached. This might exhibit an indistinct cooling effect among tested groups. Furthermore, the difference in number of firing cycles — one cycle for press-on and CAD-on technique, and three cycles for layering technique — probably generated different amounts of residual stress to enhance fracture resistance. However, it was learned that excessive residual had never occurred in such a tempering process, based on this study. This can ensure that S-cooling processes are always enhancing flexural strength for either CAD-on or press-on techniques. Contrarily, the F-cooling process seems to favor inducing residual stress for the layering technique. However, the layering technique provides less flexural strength than the CAD-on technique, which might relate with the reduction in micro-tensile bond strength when the firing cycles were increased, as supported by other studies (25,29). S-cooling aimed to minimize the thermal gradient by consistent cooling of ceramic both at the inner and outer surface below the Tg to prevent excessive occurrence of transient stresses (14,24,28). Furthermore, some studies found better outcomes for F-cooling (22,23). It was suggested that stress developed upon F-cooling could improve fracture resistance of the ceramic surface, but might generate immense tensile stress at the interfacial zone.

The microscopic investigation indicated that the fracture initiated at the veneering ceramic propagated to the interface and penetrated through the zirconia substructure until failure. The fracture patterns of CAD-on groups were rather perpendicular to the surface of zirconia, whereas the others were slightly oblique. This possibly related with the lower fracture toughness and amount of porosities in different ceramic veneering techniques, which resulted in the different paths of crack propagation. A number of porosities found in layering- and press-on groups could act as stress concentration, leading to crack propagation. The crack usually propagates along the veneer layer, where the fracture toughness is low, as well as along the core-veneer interface due to possessing a lower strain energy release rate compared to zirconia core (26). The better homogeneity of ceramic-veneering zirconia upon CAD-on technique is probably described for a reason in enhancing fracture resistance, as supported by the other study (17).

The stress generated from different veneering techniques and cooling methods can induce phase transformation. The CAD-on technique was capable of inducing higher t→m phase transformation than layering and press-on techniques. This implied that the capability of generating residual compressive stress for CAD-on was higher than other techniques, which resulted in better enhancing of flexural strength. To derive for a suitable amount of residual stress for prompt flexural strength, the appropriate ceramic veneering technique needs to be considered primarily (22). This study clearly showed that the CAD-on ceramic veneering Y-TZP effectively conferred favorable and reliable flexural strength, which was consistent with other studies (13,16,22). Although an ideal technique for ceramic-veneering Y-TZP has not been established, the selection of veneering technique must be carefully considered. In this study, the CAD-on technique was recommended to achieve favorable flexural strength of ceramic-veneered Y-TZP restoration.

## Conclusions

This investigation described the role of ceramic veneering techniques and thermal tempering processes for the flexure strength of ceramic-veneered zirconia. The experiment indicated that flexure strength of ceramic-veneered zirconia is primarily influenced by veneering technique. The CAD-on technique rendered favorable flexural strength, as it is capable of inducing residual stress to enhance fracture resistance for ceramic-veneered Y-TZP. The tempering process did not impair flexural strength. However, S-cooling tends to favor providing suitable residual stress for CAD-on and press-on techniques, whereas F-cooling seems favorable for layering techniques. Proper selection of ceramic-veneering Y-TZP is extremely crucial to assure durable fracture resistance. CAD-on was suggested as a suitable veneering technique that conferred favorable fracture resistance for ceramic-veneered Y-TZP.
